# *In Vitro* and *In Vivo* Evaluation of APX001A/APX001 and Other Gwt1 Inhibitors against Cryptococcus

**DOI:** 10.1128/AAC.00523-18

**Published:** 2018-07-27

**Authors:** Karen Joy Shaw, Wiley A. Schell, Jonathan Covel, Gisele Duboc, C. Giamberardino, Mili Kapoor, Molly Moloney, Quinlyn A. Soltow, Jennifer L. Tenor, Dena L. Toffaletti, Michael Trzoss, Peter Webb, John R. Perfect

**Affiliations:** a Amplyx Pharmaceuticals, San Diego, California, USA; b Division of Infectious Diseases, Department of Medicine, Duke University Medical Center, Durham, North Carolina, USA

**Keywords:** APX001, APX001A, Gwt1, antifungal, Cryptococcus, infection model, 1-aminobenzotriazole

## Abstract

Cryptococcal meningitis (CM), caused primarily by Cryptococcus neoformans, is uniformly fatal if not treated. Treatment options are limited, especially in resource-poor geographical regions, and mortality rates remain high despite current therapies. Here we evaluated the *in vitro* and *in vivo* activity of several compounds, including APX001A and its prodrug, APX001, currently in clinical development for the treatment of invasive fungal infections. These compounds target the conserved Gwt1 enzyme that is required for the localization of glycosylphosphatidylinositol (GPI)-anchored cell wall mannoproteins in fungi. The Gwt1 inhibitors had low MIC values, ranging from 0.004 μg/ml to 0.5 μg/ml, against both C. neoformans and C. gattii. APX001A and APX2020 demonstrated *in vitro* synergy with fluconazole (fractional inhibitory concentration index, 0.37 for both). In a CM model, APX001 and fluconazole each alone reduced the fungal burden in brain tissue (0.78 and 1.04 log_10_ CFU/g, respectively), whereas the combination resulted in a reduction of 3.52 log_10_ CFU/g brain tissue. Efficacy, as measured by a reduction in the brain and lung tissue fungal burden, was also observed for another Gwt1 inhibitor prodrug, APX2096, where dose-dependent reductions in the fungal burden ranged from 5.91 to 1.79 log_10_ CFU/g lung tissue and from 7.00 and 0.92 log_10_ CFU/g brain tissue, representing the nearly complete or complete sterilization of lung and brain tissue at the higher doses. These data support the further clinical evaluation of this new class of antifungal agents for the treatment of CM.

## Abstract (precis)

Cryptococcal meningitis (CM), caused primarily by Cryptococcus neoformans, is uniformly fatal if not treated. Treatment options are limited, especially in resource-poor geographical regions, and mortality rates remain high despite current therapies.

## INTRODUCTION

The worldwide burden of cryptococcosis is significant, with cryptococcal meningitis (CM) being the most common clinical presentation. CM's association with immune suppression during the HIV pandemic drove the annual global mortality incidence to over 1 million cases and an estimated 600,000 deaths in the early 2000s (1). An analysis of HIV-associated CM in 2014 estimated over 200,000 incident cases globally, with cryptococcosis being the second most common cause of mortality in AIDS patients (2).

The current standard-of-care regimens for CM include intravenous (i.v.) liposomal or deoxycholate amphotericin B (AMB) plus oral (p.o.) flucytosine (5FC) induction therapy for at least 2 weeks, followed by fluconazole (FLC) for a minimum of 8 weeks and then FLC suppressive therapy for 1 year (3). Clinical studies have clearly shown that rapid killing of cryptococcal cells in the central nervous system (CNS) is associated with an improved host outcome (4–6). Although AMB in combination with 5FC represents the best current fungicidal regimen, it has significant safety liabilities and is often not available in resource-limited settings where the need for effective and simple-to-use treatment is the greatest. Even in resource-available regions, such as North America and Europe, CM still occurs with a mortality rate of up to 30% in some high-risk groups (7, 8). For those patients receiving any treatment, the 1-year mortality rate from CM was 70% in low-income countries, 30% in Europe, and 20% in the United States (2). The morbidity and long-term health care costs (5 years postinfection) remain substantial (9). These data highlight several critical issues associated with CM treatment: (i) the need for additional therapies that can be used alone or in combination to reduce mortality rates, (ii) the need for a better global distribution of effective therapies, especially to resource-poor regions, (iii) the need for safer therapeutic options that are better tolerated, and (iv) ease-of-use considerations, such as all-oral options, especially where long-term maintenance therapy is required (10).

APX001 (formerly E1211) is a first-in-class small-molecule antifungal that is currently in clinical development (11, 12). APX001 is an *N*-phosphonooxymethyl prodrug which is rapidly and completely metabolized by systemic alkaline phosphatases to the active moiety, APX001A (formerly E1210) (13). Previous studies have shown that APX001A targets the Gwt1 fungal enzyme, a highly conserved inositol acylase which catalyzes an early step in the glycosylphosphatidylinositol (GPI)-anchored biosynthesis pathway (14, 15). This inhibition prevents the appropriate localization of cell wall mannoproteins, which compromises cell wall integrity, biofilm formation, germ tube formation, and fungal growth (16, 17). The PIGW protein is the closest mammalian ortholog of the fungal Gwt1 protein; however, it is not sensitive to inhibition by APX001A (16).

APX001A has broad *in vitro* activity against major fungal pathogens, including Candida, Cryptococcus, Aspergillus, Scedosporium, Fusarium, and members of the Mucorales order (18–22). Consistent with the distinct mechanism of action, it is active against azole-resistant and echinocandin-resistant strains (23). In mouse models of invasive fungal infections, administration of APX001 (or APX001A) resulted in high survival rates and reduced colony counts of fungi in the lung, kidney, and brain tissues of infected mice (13, 24–26). The latter is particularly important, given that fungal dissemination to the brain is associated with several invasive fungal infections (27).

APX001A has an extremely short half-life in mice; thus, some efficacy models of infection were initially unremarkable. 1-Aminobenzotriazole (ABT) is a well-established time-dependent nonselective suicide inhibitor of cytochrome P450 (CYP) enzymes (28). Oral administration of ABT 2 h prior to therapy with a test compound, such as antipyrine, has been shown to increase the exposure and half-life of the test compound (28, 29). In a mouse pharmacokinetic (PK) study, administration of ABT 2 h prior to APX001 dosing similarly resulted in a longer half-life and higher exposure of the active moiety, APX001A (23). As a result, lower doses and once-a-day dosing were used in Candida efficacy models, allowing for dosing regimens that result in pharmacokinetics that more closely mimic human pharmacokinetics, where phase 1 studies in healthy volunteers have shown a half-life of 2.5 days and exposures of ≥200 μg · h/ml (11, 12).

In this study, we synthesized close analogs of APX001A and evaluated their activities against Cryptococcus neoformans and Cryptococcus gattii isolates. *N*-Phosphonooxymethyl prodrugs of these molecules were synthesized by a method analogous to that used for the synthesis of APX001 (30, 31), and two of these additional prodrugs were evaluated along with APX001 in a disseminated C. neoformans infection model where ABT at 100 mg/kg of body weight was administered orally 2 h prior to therapy.

(Portions of this work were presented at IDWeek 2017, San Diego, CA [32].)

## RESULTS

### *In vitro* activity of Gwt1 inhibitors versus Cryptococcus. (i) Antifungal susceptibility profile.

APX001A and three related analogs were highly active against all 4 fungal strains evaluated (Table 1), with MIC or minimum effective concentration (MEC) values ranging from 0.004 to 0.25 μg/ml against C. neoformans, C. gattii, Candida albicans, and Aspergillus fumigatus. APX2020 and APX2041 demonstrated 4- to 8-fold lower MIC values than APX001A versus Cryptococcus, whereas APX2039 demonstrated 32-fold lower MIC values. The three analogs did not show improved activity versus C. albicans or A. fumigatus; rather, the MIC or MEC values were 1- to 8-fold higher than those of APX001A (Table 1).

**TABLE 1:**
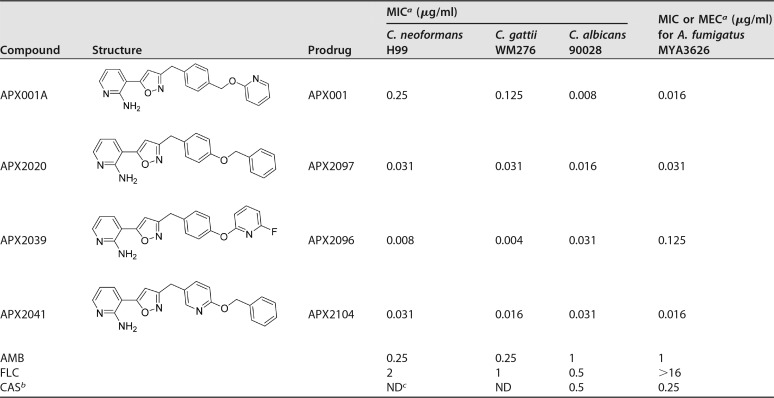
Structure and *in vitro* susceptibility profiles of Gwt1 inhibitors

^a^For the yeasts, MIC values were determined at 50% growth inhibition for FLC, caspofungin, and the APX compounds and 100% growth inhibition for AMB (47). For A. fumigatus, MEC values were determined for caspofungin and the APX compounds and MIC values were read at 100% growth inhibition for AMB and 50% growth inhibition for FLC (48).

^b^CAS, caspofungin.

^c^ND, not determined.

### (ii) Gwt1 inhibitors are synergistic with FLC.

An important consideration for developing a new treatment for CM is the potential for inclusion in combination therapy. We evaluated the synergy of APX001A and APX2020 in combination with FLC using standard microtiter dilution techniques (33). Synergy (fractional inhibitory concentration index [FICI] values < 0.5) was observed for both compounds against C. neoformans H99: the FICI was 0.37 for both FLC-APX001A and FLC-APX2020. Importantly, no antagonism was observed.

### (iii) Activity of Gwt1 inhibitors versus FLC-susceptible and FLC-nonsusceptible/resistant strains.

The activities of APX001A, APX2020, APX2039, APX2041, AMB, and FLC were examined against a collection of FLC-susceptible and FLC-nonsusceptible/resistant (MIC ≥16 μg/ml) strains of C. neoformans and C. gattii. APX2039 was the most active compound tested, with MIC values ranging from 0.004 to 0.031 μg/ml against all 18 strains tested, followed by APX2041 (MIC range, 0.016 to 0.125 μg/ml), APX2020 (MIC range, 0.031 to 0.25 μg/ml), and APX001A (MIC range, 0.125 to 0.5 μg/ml) (Table 2). Consistent with different mechanisms of action, the activities of the APX compounds as well as AMB were unchanged for FLC-resistant strains DUMC 118.00 and RSA-MW-3615 relative to susceptible strains (Table 2). FLC-resistant C. neoformans DUMC 158.03 demonstrated somewhat higher MIC values for the four APX compounds as well as AMB, suggesting that additional non-target-based mutations may be present in this strain.

**TABLE 2:** Activity of Gwt1 inhibitors versus FLC-susceptible and FLC-nonsusceptible/resistant strains of Cryptococcus

Species	Isolate	MIC (μg/ml)					
		APX001A	APX2020	APX2039	APX2041	AMB	FLC
C. neoformans	H99	0.125	0.031	0.004	0.031	0.25	1
C. neoformans	DUMC 118.00	0.25	0.063	0.016	0.063	0.25	64
C. neoformans	DUMC 158.03	0.25	0.25	0.031	0.125	1	32
C. neoformans	MYA-4564	0.125	0.063	0.004	0.016	0.25	4
C. neoformans	MYA-4565	0.5	0.25	0.031	0.125	0.125	1
C. neoformans	MYA-4566	0.25	0.125	0.008	0.063	0.25	2
C. neoformans	MYA-4567	0.25	0.063	0.016	0.031	0.25	1
C. neoformans	14116	0.125	0.031	0.004	0.016	0.25	4
C. neoformans	76484	0.125	0.063	0.004	0.016	0.25	4
C. gattii	RSA-MW-3615	0.125	0.031	0.004	0.016	0.25	64
C. gattii	MYA-4877	0.25	0.063	0.008	0.016	0.25	4
C. gattii	MYA-4093	0.5	0.125	0.016	0.125	0.25	2
C. gattii	MYA-4094	0.25	0.25	0.016	0.063	0.25	2
C. gattii	MYA-4560	0.25	0.063	0.008	0.016	0.063	1
C. gattii	MYA-4561	0.5	0.125	0.016	0.031	0.25	4
C. gattii	MYA-4562	0.25	0.125	0.016	0.031	0.25	2
C. gattii	MYA-4563	0.5	0.125	0.016	0.031	0.125	4
C. gattii	MYA-4560	0.25	0.063	0.008	0.016	0.063	1
Geometric mean		0.241	0.085	0.010	0.034	0.215	3.564
MIC_90_		0.5	0.25	0.031	0.125	0.25	64

### *In vivo* activity of Gwt1 inhibitors versus C. neoformans. (i) Efficacy of APX001 alone and in combination with FLC in a murine model of cryptococcal meningitis.

The efficacies of APX001 and FLC were evaluated in a well-established mouse CM model (34). Since Cryptococcus infections can be hematogenously disseminated to other organs, the numbers of CFU in both lung and brain tissue were evaluated. Male CD-1 mice were infected with 5.9 × 10^4^ CFU of C. neoformans strain H99 via lateral tail vein injection. Mice were assigned to four groups (*n* = 10), consisting of (i) treatment with APX001, (ii) treatment with APX001 plus FLC, (iii) treatment with FLC, or (iv) no treatment as a control. Treatment was initiated within 1 h after infection. APX001 was administered by oral gavage at a dose of 390 mg/kg thrice daily (TID), with each dose being administered roughly 8 h apart. ABT was not used in this model; thus, dosing of APX001 TID was necessitated by the short half-life of APX001A in mice (1.40 to 2.75 h) (35). FLC (2 mg/ml; Sagent Pharmaceuticals, Schaumburg, IL) was administered at a dose of 80 mg/kg/day intraperitoneally (i.p.).

**FIG 1:**
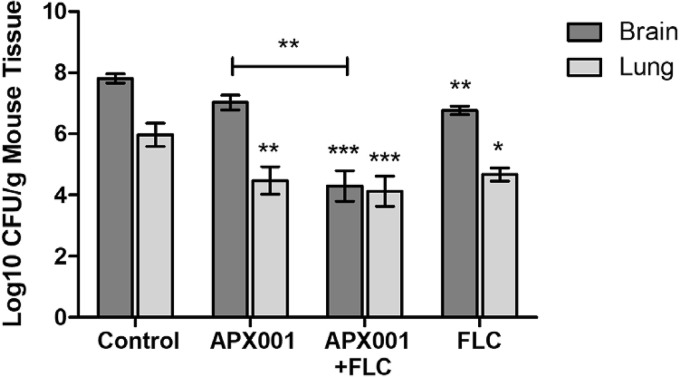
Efficacy of APX001 alone and in combination with FLC in a murine model of C. neoformans meningitis. Brain and lung burdens in mice were measured at 7 days postinfection with C. neoformans H99. Male CD-1 mice were infected with 5.9 × 10^4^ CFU of C. neoformans strain H99 via the lateral tail vein. Treatment was initiated at about 1 h postinfection and continued for 7 days. APX001 was administered by oral gavage at a dose of 390 mg/kg thrice daily, with each dose being administered roughly 8 h apart. FLC (2 mg/ml; Sagent Pharmaceuticals, Schaumburg, IL) was administered at a dose of 80 mg/kg/day i.p. once daily. Fungal burden data were log_10_ transformed and evaluated using Kruskal-Wallis tests with Dunn's multiple-comparison test for *post hoc* analysis (Prism software, v5; GraphPad Software, Inc., San Diego, CA). A *P* value of ≤0.05 was considered statistically significant (*, *P* ≤ 0.05; **, *P* ≤ 0.01; ***, *P* ≤ 0.001). The 95% confidence interval (CI) is presented.

The mean brain and lung tissue counts in untreated control mice were 7.81 ± 0.19 and 5.97 ± 0.47 log_10_ CFU/g, respectively (Fig. 1). Significant differences (*P* < 0.05) in the lung tissue fungal burden were observed between all treatment groups (APX001, FLC, and APX001 plus FLC) and the untreated control group (Fig. 1). In lung, the reductions in fungal burden compared to that in the untreated control were similar for all three treatments groups (APX001, 1.50 log_10_ CFU/g; FLC, 1.30 log_10_ CFU/g; combined therapy, 1.84 log_10_ CFU/g), with no statistically significant differences between the treatment groups being found (Fig. 1).

In brain, mice treated with APX001 demonstrated a reduction of the fungal burden of 0.78 log_10_ CFU/g compared with that in the control group, but this difference was not statistically significant. However, significant reductions in the fungal burden compared with that in the control group were observed for FLC alone (1.04 log_10_ CFU/g) and the combination of APX001 and FLC (3.51 log_10_ CFU/g) (*P* < 0.01 and *P* < 0.001, respectively) (Fig. 1).

### (ii) Effect of ABT on the PK of APX compounds.

The pharmacokinetics (PK) of APX001 in combination with ABT have been previously assessed in female CD-1 mice (23). In that study, oral administration of 100-mg/kg ABT 2 h prior to administration of APX001 (i.p.) dramatically extended the half-life of the active moiety, APX001A, from 1.3 to 8.8 h, resulting in a 9-fold increase in the area under the curve (AUC) (23), which has previously been shown to be the driver of efficacy (35).

In a similar fashion, the PK of APX001A, APX2039, APX2020, and APX2041 were compared in male CD-1 mice after the administration of 26 mg/kg of the corresponding prodrug (APX001, APX2096, APX2097, APX2104) either by the oral route or by i.p. injection (Table 3). In half of the cohorts, 100-mg/kg ABT was administered 2 h prior to compound administration. Although the AUC values of the analytes differed up to 4-fold after oral administration of the four prodrugs, the addition of ABT resulted in similar exposures for APX001A, APX2020, and APX2041. The resulting exposure for APX2039 was approximately 2-fold higher than that for the three other compounds evaluated. The addition of ABT resulted in 8.6- to 15-fold increased exposure after oral administration of the prodrugs.

**TABLE 3:** Exposures of Gwt1 inhibitors following oral or i.p. dosing of prodrugs in the presence or absence of 100 mg/kg ABT pretreatment^*a*^

Prodrug	Analyte	Avg AUC^*b*^ (μg · h/ml) resulting from 26-mg/kg dose				Ratio of AUC with ABT/AUC without ABT	
		p.o. dosing	i.p. dosing	p.o. dosing + ABT	i.p. dosing + ABT	p.o. dosing	i.p. dosing
APX001	APX001A	2.76 ± 0.23	4.36 ± 0.11	41.50 ± 8.09	24.28 ± 17.74	15.0	5.6
APX2096	APX2039	10.66 ± 0.48	11.75 ± 1.83	91.28 ± 20.75	97.25 ± 12.61	8.6	8.3
APX2097	APX2020	4.49 ± 2.32	4.31 ± 0.96	41.94 ± 6.41	35.61 ± 28.22	9.3	8.3
APX2104	APX2041	3.49 ± 0.27	4.68 ± 0.73	49.92 ± 10.34	72.62 ± 9.07	14.3	15.5

a p.o., oral; i.p., intraperitoneal.

b Time course for the experiment: 0.083 0.5, 2, 4, 8, and 24 h postdose of 26 mg/kg prodrug (*n* = 3 per time point). AUC is the area under the curve of the analyte, calculated from time zero to the time of the last measurable concentration.

When the prodrugs were administered i.p., similar exposures were obtained for the analytes APX001A, APX2020, and APX2041, while APX2039 demonstrated an approximately 2-fold higher AUC than the other compounds evaluated (Table 3). The addition of ABT prior to i.p. drug administration increased exposures from 5.6- to 15.5-fold. Intraperitoneal dosing was chosen as the route of administration for APX001, APX2096, and APX2097 in the CM model.

### (iii) Efficacy of APX compounds in a murine model of cryptococcal meningitis when dosed in the presence of the pan-CYP inhibitor ABT.

**FIG 2:**
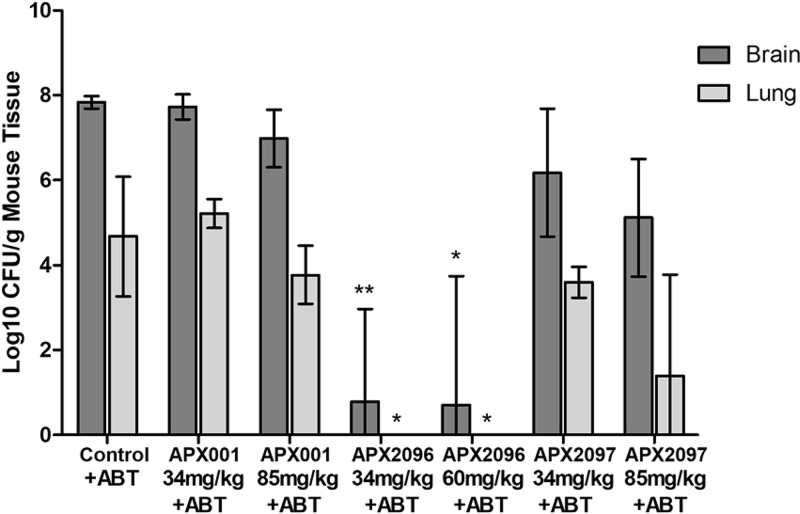
Efficacy of APX compounds in a murine model of cryptococcal meningitis in conjunction with ABT administration. ABT at 100 mg/kg was administered orally to male CD-1 mice at 2 h prior to each APX compound dose. Mice were injected with 6.9 × 10^4^ CFU of C. neoformans strain H99 per mouse via the lateral tail vein at time zero. Treatment with the APX compound was initiated at about 1 h postinfection by i.p. administration and continued for 7 days, with 100-mg/kg ABT being administered orally 2 h prior to each APX compound dose. The correction factors for the prodrug potencies versus the potencies of the active moieties were similar enough such that the same factor (1.3) was used for all APX compounds. Acute toxicity was observed on day 1 for APX2096 and on day 6 for APX2097, and therefore, the dose was reduced to 60 mg/kg for days 2 to 7 (APX2096) and day 7 (APX2097) for the surviving mice. The final numbers of surviving mice were 4 for the controls, 5 for APX001-treated mice, 3 to 5 for APX2096-treated mice, and 4 or 5 for APX2097. Fungal burden data were log_10_ transformed and evaluated using Kruskal-Wallis tests with Dunn's multiple-comparison test for *post hoc* analysis (Prism software, v5; GraphPad Software, Inc., San Diego, CA). A *P* value of ≤0.05 was considered statistically significant (*, *P* ≤ 0.05; **, *P* ≤ 0.01). The 95% confidence interval (CI) is presented.

In a preliminary experiment, the efficacies of APX001, APX2096, and APX2097 were evaluated in the disseminated model of CM (*n* = 5 mice/cohort). ABT at 100 mg/kg was administered orally to male CD-1 mice 2 h prior to APX compound administration. Mice were injected with 6.9 × 10^4^ CFU of C. neoformans strain H99 per mouse via the lateral tail vein at time zero. Treatment with each prodrug was initiated at about 1 h postinfection by i.p. administration and continued daily for 7 days, with 100-mg/kg ABT being administered orally 2 h prior to each APX compound dose. The mean brain and lung counts in untreated control mice were 7.83 ± 0.09 and 4.67 ± 0.88 log_10_ CFU/g, respectively (Fig. 2).

In lung, neither the 34-mg/kg nor the 85-mg/kg dose of APX001 achieved a statistically significant reduction in log_10_ CFU/g of tissue compared with that in the untreated control. Of note is that 390-mg/kg APX001 dosed orally TID (Fig. 1) resulted in higher AUC values than 85-mg/kg APX001 dosed i.p. once daily (QD) with ABT (Fig. 2); thus, better efficacy in lung was observed with APX001 monotherapy, as shown in Fig. 1. In lung, administration of 60-mg/kg or 34-mg/kg APX2096 reduced the tissue burdens to below the limit of detection (approximately 4.67 log_10_ CFU/g lung tissue). For APX2097, the reduction in the number of CFU in lung was 3.28 log_10_ CFU/g (85 mg/kg QD) and 1.07 log_10_ CFU/g (34 mg/kg QD).

In brain, administration of 60-mg/kg or 34-mg/kg APX2096 resulted in a reduction of 7.13 and 7.05 log_10_ CFU/g brain tissue, respectively. For APX2097, the reduction of the number of CFU in brain was 2.72 log_10_ CFU/g (85 mg/kg QD) and 1.66 log_10_ CFU/g (34 mg/kg QD). Administration of 85-mg/kg APX001 demonstrated a modest reduction in the fungal burden (0.85 log_10_ CFU/g), which did not, however, achieve statistical significance.

A dose-response study was performed with APX2096 and APX2097 to confirm the observed activity. In this study, QD doses of 7.5, 20, and 60 mg/kg were evaluated in conjunction with the administration of ABT. A 60-mg/kg dose QD without ABT was also evaluated as a control.

**FIG 3:**
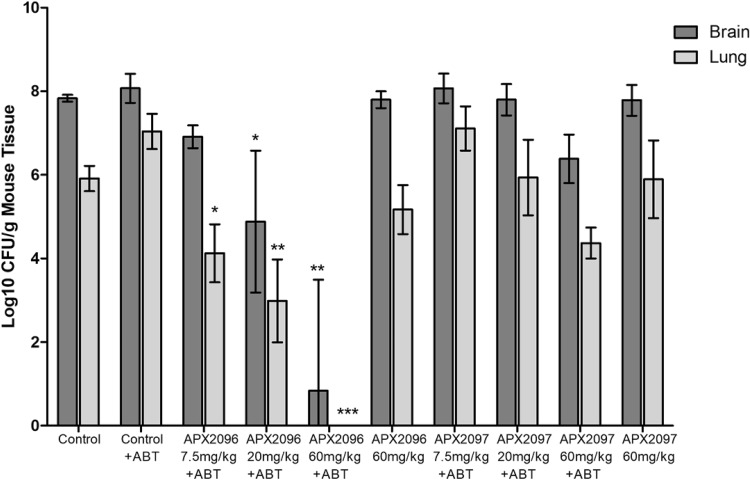
Dose-response of APX2096 and APX2097 in a murine model of cryptococcal meningitis in conjunction with ABT administration. ABT at 100 mg/kg was administered orally to male CD-1 mice at 2 h prior to each APX compound dose. Mice were injected with 4.8 × 10^4^ CFU C. neoformans strain H99 per mouse via the lateral tail vein at time zero. Treatment with the APX compounds was initiated at 1 h postinfection by i.p. administration and continued for 7 days, with 100-mg/kg ABT being administered orally 2 h prior to each APX compound dose. The correction factors for prodrug potencies versus the potencies of the active moieties were similar enough such that the same factor (1.3) was used for all APX compounds. Fungal burden data were log_10_ transformed and evaluated using Kruskal-Wallis tests with Dunn's multiple-comparison test for *post hoc* analysis (Prism software, v5; GraphPad Software, Inc., San Diego, CA). For analysis of the fungal burden, the control mice that received ABT were used for comparisons to APX compound-treated groups receiving ABT, and the untreated control mice were used for comparisons to APX compound-treated groups that did not receive ABT. A *P* value of ≤0.05 was considered statistically significant (*, *P* ≤ 0.05; **, *P* ≤ 0.01; ***, *P* ≤ 0.001). The 95% confidence interval (CI) is presented.

The mean counts in untreated control mouse tissue were 7.83 ± 0.07 log_10_ CFU/g (brain tissue) and 5.91 ± 0.24 log_10_ CFU/g (lung tissue) (Fig. 3). Control animals, which received daily doses of 100-mg/kg ABT without an APX compound, had values of 8.07 ± 0.28 log_10_ CFU/g (brain tissue) and 7.04 ± 0.34 log_10_ CFU/g (lung tissue). It is not clear why in this experiment the ABT-treated mice demonstrated a 1-log increase in the CFU counts in lung tissue, whereas similar counts were obtained in brain tissue.

Both APX2096 and APX2097 demonstrated a dose-response in the reduction of the number of log_10_ CFU/g of brain and lung tissue when ABT was utilized. Cohorts which received 60 mg/kg/day of APX compounds without ABT showed either a numerical but nonsignificant reduction in the lung burden of 0.74 log_10_ CFU/g (APX2096) or no reductions in the number of log_10_ CFU/g of mouse tissue, consistent with a shorter half-life and lower exposure.

For APX2096 with ABT, the dose-dependent reductions in counts ranged from 5.91 to 1.79 log_10_ CFU/g for lung tissue and from 7.00 to 0.92 log_10_ CFU/g for brain tissue. All ABT-plus-APX2096 treatment cohorts demonstrated reductions in fungal lung burden that were statistically significantly different from those for the ABT-administered control group (*P* ≤ 0.05). The two highest ABT-plus-APX2096 dosing levels also showed reductions in brain tissue fungal burden, ranging from 6.99 to 2.95 log_10_ CFU/g (*P* ≤ 0.05).

For APX2097, dose-dependent changes in the fungal burden ranged from a reduction of 1.55 log_10_ CFU/g to an increase of 1.20 log_10_ CFU/g for lung and from a reduction of 1.45 log_10_ CFU/g to an increase of 0.24 log_10_ CFU/g for brain. However, none of these reductions reached statistical significance compared with the results for the ABT-administered control group. Statistical significance was also not achieved when these cohorts were evaluated versus the no-ABT vehicle control group.

The results of the dose-response experiment were consistent with the preliminary finding that APX2096 demonstrated nearly complete or complete sterilization of lung and brain tissue at doses of 34 and 60 mg/kg (plus ABT).

### (iv) Analysis of AUC values versus the change in the number of log_10_ CFU per gram of tissue.

The three compounds evaluated in the efficacy model had MIC values for the infecting strain (C. neoformans H99) that differed by 8- to 32-fold: for APX001A, 0.25 μg/ml; for APX2020, 0.031 μg/ml; and for APX2039, 0.008 μg/ml (Table 1). The data in Table 3 show that AUC values after i.p. dosing (plus ABT) ranged from 24.3 to 97.3 μg · h/ml, representing a 4-fold difference. To understand the influence of AUC-versus-MIC differences, we assessed the magnitude of changes in the number of log_10_ CFU/g of tissue across the three experiments.

AUC values across the three experiments for APX001 (with or without ABT) ranged from 7.0 μg · h/ml (7.5-mg/kg APX001 QD plus ABT) to 196.3 μg · h/ml (390 mg/kg TID). At an AUC of 196.3 μg · h/ml, a modest but significant reduction in the lung burden was observed (1.5 log_10_ CFU/g). Lower AUC values were not efficacious. AUC values ranged from 10.0 to 116.4 μg · h/ml for APX2097 and from 27 to 224.3 μg · h/ml for APX2096. We compared the efficacy of the three compounds at a dose that gave rise to AUC values of approximately 80 µg · h/ml. In the presence of ABT, doses of 20-mg/kg APX2096, 60-mg/kg APX2097, and 80-mg/kg APX001 resulted in very similar AUC values of 74.8, 82.1, and 79.4 μg · h/ml, respectively. However, the reductions were 2.95, 1.45, and 0.85 log_10_ CFU/g, respectively, in brain and 3.69, 1.55, and 0.9 log_10_ CFU/g, respectively, in lung. Thus, despite the same AUC values for the 3 compounds, better efficacy was associated with lower MIC values (0.008 μg/ml, 0.031 μg/ml, and 0.25 μg/ml, respectively), suggesting that improved microbiological activity largely accounts for improved efficacy.

### (v) Efficacy of APX2096 and AMB in a delayed-treatment model of cryptococcal meningitis.

A delayed-treatment model was used to compare the efficacy of once-daily treatment using 60-mg/kg (i.p.) APX2096 or 3-mg/kg (i.p.) AMB compared with that of the vehicle control (i.p. 5% dextrose). As in the previous mouse model, 100-mg/kg ABT (p.o.) was administered 2 h prior to each APX2096 dose (*n* = 5 mice/cohort). Infection was initiated on day 1, and treatment was initiated 24 h later (day 2) rather than 1 h later. Treatments were administered for 7 days (the final dose was administered on day 8), and mice were sacrificed on day 9 for CFU enumeration.

**FIG 4:**
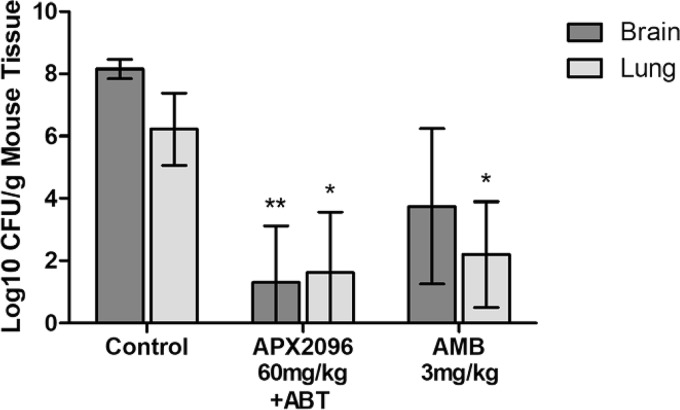
Efficacy of APX2096 and AMB in a delayed-treatment model of cryptococcal meningitis. Male CD-1 mice were infected with 5.4 × 10^4^ CFU per mouse via lateral tail vein injection. Treatment with 60-mg/kg APX2096, 3-mg/kg AMB, or the vehicle control was initiated 24 h after infection and continued daily for 7 days. A dose of 100-mg/kg ABT was administered orally 2 h prior to each APX2096 dose. Mice were sacrificed 24 h after the last dose, and the brain and left lung were homogenized and cultured for quantitative determination of the tissue burden (number of CFU per gram of tissue). Fungal burden data were log_10_ transformed and evaluated using Kruskal-Wallis tests with Dunn's multiple-comparison test for *post hoc* analysis (Prism software, v5; GraphPad Software, Inc., San Diego, CA). A *P* value of ≤0.05 was considered statistically significant (*, *P* ≤ 0.05; **, *P* ≤ 0.01). The 95% confidence interval (CI) is presented.

The mean counts in untreated control mice were 8.15 ± 0.24 log_10_ CFU/g (brain tissue) and 6.22 ± 0.93 log_10_ CFU/g (lung tissue) (Fig. 4). Both APX2096 and AMB demonstrated a statistically significant reduction of the lung tissue fungal burden (4.59 and 4.02 log_10_ CFU/g, respectively) compared with that in the untreated control group (*P* ≤ 0.05). APX2096 also showed a reduction of 6.84 log_10_ CFU/g in brain tissue compared with that in the untreated control group, which was significant (*P* ≤ 0.01). These data are very similar to the reductions observed in the 60-mg/kg APX2096-plus-ABT cohort (Fig. 3), demonstrating the reproducibility of these findings. Although AMB demonstrated a 4.40-log_10_ CFU/g reduction in brain tissue, this did not meet statistical significance.

## DISCUSSION

The prodrug APX001 is a first-in-class, i.v. and orally available broad-spectrum antifungal agent in clinical development for the treatment of life-threatening invasive fungal infections. In this study and in previous studies, APX001A, the active moiety of APX001, has been shown to have good *in vitro* activity against C. neoformans (19). Two additional compounds, APX2020 and APX2039, demonstrated 8- to 32-fold improved anti-C. neoformans H99 activity, with MIC values of 0.031 and 0.008 μg/ml, respectively. This difference in activity was also seen against a larger panel of 18 isolates for which MIC_90_ values were 0.5 μg/ml (APX001A), 0.25 μg/ml (APX2020), and 0.031 μg/ml (APX2039) (Table 2). These values compare favorably to those for other drugs in clinical use for the treatment of CM. In a global study that evaluated antifungal activity versus 46 strains of C. neoformans, MIC_90_ values for the azoles ranged from 0.06 μg/ml (isavuconazole) to 4 μg/ml (FLC), whereas the echinocandins were largely inactive, with MIC_90_ values being ≥16 μg/ml (36). Similarly, in a study of U.S. isolates, MIC_90_ values for C. neoformans were 2 μg/ml for AMB and 8 μg/ml for flucytosine, with only itraconazole (MIC_90_, 0.125 μg/ml) and ketoconazole (MIC_90_, 0.06 μg/ml) demonstrating low MIC_90_ values (37).

FLC nonsusceptibility or resistance has been observed clinically in both C. neoformans and C. gattii (38–40), although Clinical and Laboratory Standards Institute (CLSI) breakpoints have not been definitively established. Several studies have shown a correlation between high FLC MIC values (≥16 μg/ml) and poor clinical outcomes (38, 39). In a single-center study of 89 C. neoformans isolates recovered between 2001 and 2012 in southern Taiwan, 34% were FLC nonsusceptible (MIC values ≥ 16 μg/ml) (41). In the current study, we examined a collection of clinically isolated FLC-susceptible and FLC-nonsusceptible/resistant strains of C. neoformans and C. gattii. Consistent with a different mechanism of action, the potency of the four Gwt1 compounds relative to that of FLC was maintained, although one strain (DUMC 158.03) had higher MIC values for the APX compounds as well as AMB, suggesting that additional non-target-based mutations may be present in this strain. Despite the elevated MIC values for this strain, it is anticipated that appropriate clinical exposures may still be achieved for coverage of these types of strains.

Standard microtiter checkerboard dilution experiments demonstrated that both APX001A and APX2020 are synergistic with FLC versus C. neoformans H99. These data are consistent with previous reports of APX001A synergy with FLC against 9 of 10 Candida tropicalis strains and 2 of 20 strains of C. albicans (24). Importantly, no antagonism was observed. Improved activity in combination with FLC was also observed in the CM mouse model. Monotherapy of APX001 or FLC resulted in a reduction of 0.78 and 1.04 log_10_ CFU/g brain tissue compared with the fungal burden in the untreated controls, whereas the combination of APX001 and FLC resulted in a reduction of 3.52 log_10_ CFU/g brain tissue compared to the fungal burden in the controls. This combination therapy was significantly more active in the reduction of the fungal burden in the brain than monotherapy with APX001. Importantly, APX001 demonstrated >90% bioavailability in phase 1 clinical trials (11, 12), laying the foundation for an all-oral APX001-plus-FLC regimen for human cryptococcal infections.

Many molecules are discarded in early-stage programs due to poor pharmacokinetic profiles in mice. Metabolic instability resulting in a short half-life (e.g., 1 h) necessitates multiple doses per day at relatively high concentrations to maintain the drug levels required for efficacy. These findings may not be recapitulated in phase 1 clinical studies, as is the case for APX001, where a very long half-life was observed in humans (2.5 days) (11, 12) but a short half-life (1.3 to 2.5 h) was observed in mice (35). Other attempts at improving the poor pharmacokinetics in mice, such as the administration of grapefruit juice prior to dosing, were not successful for APX001 (unpublished observations).

ABT has been used to increase the exposure of drugs in other therapeutic animal models; however, the use of ABT for improving efficacy in infectious disease models has not been widespread. Two studies utilized ABT in short-term models, where the numbers of log_10_ CFU/g of tissue were examined after 24 to 48 h. The antibacterial efficacy of experimental adenosine analogs targeting DNA ligase was evaluated at 24 h postinfection in a thigh model in which mice received a single dose of 100-mg/kg ABT 2 h prior to infection to reduce the high hepatic clearance (42). The efficacy of APX001 was examined after ABT administration in disseminated Candida infection models, where C. albicans kidney burdens were reduced 6.0 ± 0.1 log_10_ CFU/g kidney tissue after 48 h (23). Since efficacy models can require treatments lasting 7 days or longer, the ability to maintain good drug exposures by administration of ABT over the treatment period is important. One study in mice examined the pharmacokinetic parameters of antipyrine administered as a 14-day continuous infusion of 20 or 60 mg ABT by an Alzet osmotic pump (43). In that study, AUC values increased 3- to 4-fold when antipyrine was dosed intravenously (i.v.) and 8- to 10-fold after oral administration, demonstrating the feasibility of long-term ABT administration. Here we show that 7 days of daily administration of 100-mg/kg ABT 2 h prior to APX compound treatment dramatically increased the efficacy of three Gwt1 inhibitors. Pharmacokinetic studies demonstrated that ABT increased exposures 5.6- to 15.5-fold when APX molecules were dosed orally and 8.6- to 15-fold when APX molecules were dosed i.p. These data support the use of ABT in infectious disease animal models for analysis of both clinical candidates and early-discovery molecules when proof-of-concept data are required.

Clinical studies have clearly shown that rapid killing of cryptococcal cells in the CNS is associated with an improved host outcome (4–6). Strikingly, our animal model data provide evidence of effective brain penetration, one of the key factors in the choice of a drug for the treatment of CM. These data are consistent with the results of [^14^C]APX001 distribution studies, which demonstrated significant radioactivity in tissues associated with invasive fungal infections, including brain tissue (44), whereas poor CNS penetration has been observed for the echinocandins (45). Notably, APX2096 significantly reduced the lung and brain tissue fungal burden in a murine CM model, where in past experience, only AMB has shown a similar reduction in the number of CFU in this model (J. R. Perfect, personal observations). In the current study, the activity of APX2096 was at least comparable to or better than that of AMB in a delayed-treatment model. Thus, an oral agent with the potential to kill yeasts rapidly in the CNS of a host, such as APX2096, is of significant interest. We hypothesize that the outstanding anticryptococcal activity of analogs such as APX2096 in this murine model represents a combination of extremely potent direct antifungal activity against Cryptococcus and favorable CNS penetration. Further pharmacodynamic studies will be performed after the optimal Gwt1 inhibitor is identified.

Phase 1 clinical studies have demonstrated >90% bioavailability for APX001, and both oral and i.v. formulations are in development (11, 12). Similarly, mouse pharmacokinetic studies of all three prodrugs suggest that exposures after oral dosing are equivalent to or better than those of APX001, raising the possibility that other Gwt1 inhibitors can also be developed for oral administration, enabling new and rapidly effective all-oral regimens for the treatment of CM either alone or in combination with FLC.

## MATERIALS AND METHODS

### Synthesis of Gwt1 inhibitors and *N*-phosphonooxymethyl prodrugs.

The synthesis of APX001A, APX2020, and APX2041 has been previously described (46). The synthesis of APX2039 is shown in Fig. S1 in the supplemental material. Previous studies have shown that introduction of an *N*-phosphonooxymethyl prodrug moiety at the aminopyridine group leads to a substantial increase in the solubility of the drug substance (30). Following documented procedures, we synthesized *N*-phosphonooxymethyl prodrugs of three Gwt1 inhibitors from our scale-up efforts (Fig. S3 and S5).

### Isolates tested.

C. neoformans strains H99, DUMC 118.00, and DUMC 158.03 and C. gattii strains R272 and RSA-MW-3515 were obtained from Duke University. C. albicans 90028, A. fumigatus MYA3626, C. neoformans 14116, C. neoformans 76484, and the pathogenic Cryptococcus reference strains panel (ATCC MP-11) were obtained from the American Type Culture Collection (ATCC; Manassas, VA, USA). The ATCC MP-11 panel consists of strains representing eight molecular types and three subtypes of C. neoformans and C. gattii.

### Antifungal agents.

All drug stock solutions were prepared at 10 mg/ml in 100% dimethyl sulfoxide (DMSO), and aliquots were stored at −20°C. The following drugs were tested: AMB (VWR, Radnor, PA, USA), FLC (Alfa Aesar, Tewksbury, MA, USA, or Sagent Pharmaceuticals, Schaumburg, IL), caspofungin (Sigma, St. Louis, MO, USA), and APX001A, APX2020 APX2039, and APX2041 (Amplyx Pharmaceuticals).

For pharmacokinetic and efficacy studies, the prodrugs APX001, APX2096, APX2097, and APX2104 (Amplyx Pharmaceuticals) were used. APX001, the *N*-phosphonooxymethyl prodrug, is soluble in water. On adding APX001 to water, the pH is less than 7.0. Sodium hydroxide was added to bring the pH back to a neutral range, maintain solubility, and allow dosing of the formulated material. The prodrugs APX2096, APX2097, and APX2104 were formulated similarly to enable oral and i.p. dosing of compounds for pharmacokinetic and efficacy studies. Final prodrug solutions were in 5% dextrose and dosed orally (p.o.) or i.p. on a per gram of mouse body weight daily basis. A 10-mg/ml solution of ABT (Fisher Scientific, Hampton, NH) in water was administered orally 2 h prior to infection as 10 μl per gram of mouse body weight, resulting in a dose of 100 mg/kg.

### Antifungal susceptibility testing.

To establish the antimicrobial activity of the APX001A analogs, broth microdilution susceptibility testing was performed according to Clinical and Laboratory Standards Institute (CLSI) guidelines M27-A3 (47) for yeasts and M38-A2 for molds (48). APX001A and analogs were first diluted in DMSO to obtain intermediate dilutions. These were further diluted in microtiter plates to obtain a final concentration of 2 to 0.002 μg/ml. One microliter of DMSO was added to the no-drug control wells. The solutions were mixed on a plate shaker for 10 min, and the plates were incubated at 35°C for 40 to 48 h (C. albicans, A. fumigatus) and 72 h (C. neoformans). The minimum concentration that led to a 50% reduction in fungal growth compared to that for the control (determined with the aid of a reading mirror) was determined as the MIC for C. albicans and C. neoformans. The minimum concentration that led to the shortening of hyphae compared to the hyphal growth in DMSO control wells was determined as the minimum effective concentration (MEC) for A. fumigatus (as read for the echinocandins). The use of the MIC and MEC endpoints for APX001A (formerly E1210) against yeasts and molds, respectively, has been described previously (19–21). For the cryptococcal synergy studies, APX001A and APX2020 MIC values were read at 50% inhibition.

### Pharmacokinetic analysis.

Single-dose PK experiments were performed in healthy male CD-1 mice following i.p. or oral dosing of 26 mg/kg of the prodrugs APX001, APX2096, APX2097, and APX2104. In half of the cohorts, mice received a single oral dose of 100-mg/kg ABT at 2 h prior to prodrug dosing. Plasma was collected at 0.083, 0.5, 2, 4, 8, and 24 h postdose (*n* = 3 per time point). The area under the curve (AUC) was calculated from time zero to the time of the last measurable concentration. The active metabolite concentrations in plasma (APX001A, APX2039, APX2020, and APX2041) were determined by liquid chromatography-tandem mass spectrometry. PK parameters were determined using Phoenix WinNonlin (v7.0) software and a noncompartmental model. Samples with concentrations that were below the limit of quantification (0.5 or 1 ng/ml) were not used in the calculation of averages.

### Cryptococcal meningitis model.

C. neoformans strain H99 was grown in yeast extract-peptone-dextrose broth at 30°C on a shaker (220 rpm) for 24 h, centrifuged (1,980 relative centrifugal force), and washed twice in phosphate-buffered saline (PBS), resuspended in PBS, and quantified by hemacytometric count. Male CD-1 mice were infected with ∼5 × 10^4^ CFU per mouse via lateral tail vein injection of 100 μl. Mice were weighed, and treatment was begun within 1 h after infection. Treatments were administered daily for 7 days. Mice were weighed daily and observed for acute and chronic adverse symptoms. Mice were sacrificed on day 8, and the brain and left lung were homogenized and cultured for quantitative determination of the tissue burden (number of CFU per gram of tissue). Tissues were homogenized for 25 s in 1 ml phosphate-buffered saline using two 6.5-mm steel beads and a Mini-Beadbeater 16 apparatus (Biospec Products, Inc., Bartlesville, OK) and serially diluted in 10-fold steps. Aliquots (100 μl) of homogenate were plated and incubated for 3 to 7 days at 37°C. Fungal burden data were log_10_ transformed and evaluated using Kruskal-Wallis tests with Dunn's multiple-comparison test for *post hoc* analysis (Prism software, v5; GraphPad Software, Inc., San Diego, CA). A *P* value of ≤0.05 was considered statistically significant.

### Delayed-treatment model.

The delayed-treatment model was similar to the cryptococcal meningitis model with the following exceptions: (i) male CD-1 mice were infected with 5.4 × 10^4^ CFU per mouse via lateral tail vein injection of 100 μl; (ii) treatment was initiated at 24 h after infection and continued daily for 7 days, with 100-mg/kg ABT (p.o.) being administered 2 h prior to each APX2096 dose; and (iii) mice were sacrificed at 24 h after the last dose, and brain and left lung were homogenized and cultured for quantitative determination of the tissue burden (number of CFU per gram of tissue). Fungal burden data were log_10_ transformed and evaluated using Kruskal-Wallis tests with Dunn's multiple-comparison test for *post hoc* analysis (Prism software, v5; GraphPad Software, Inc., San Diego, CA). A *P* value of ≤0.05 was considered statistically significant.

### Animal use and care.

All animal-related study procedures were compliant with the Animal Welfare Act, the *Guide for the Care and Use of Laboratory Animals* (49), and the Office of Laboratory Animal Welfare.

## Supplementary Material

Supplemental file 1
