# Supplementary material for: In Vitro and In Vivo Evaluation of APX001A/APX001 and Other Gwt1 Inhibitors against Cryptococcus
Source: Antimicrob Agents Chemother. 2018 Jul 27;62(8):e00523-18. doi: 10.1128/AAC.00523-18 (PMC6105804; doi:10.1128/AAC.00523-18)
Supplement: Supplemental file 1 [file zac008187373s1.pdf]

Supplementary Data

General remarks. All ^{1}H NMR and ^{31}P NMR were recorded with a Bruker NMR spectrometer (^{1}H 500MHz, ^{31}P 200MHz) at room temperature in the solvent indicated. Flash chromatography was performed using a Biotage Isolera One and pre-packed columns (Snap®-Ultra). ESI mass spectra were recorded in ES+ mode on a PE SCIEX API 150EX Mass Spectrometer.

Fig. S1. Synthesis of 3-(3-(4-((6-fluoropyridin-2-yl)oxy)benzyl)isoxazol-5-yl)pyridin-2-amine (APX2039) *(S1)

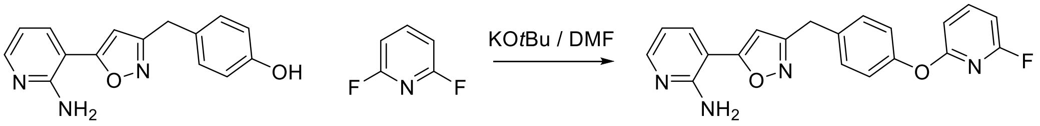

Manufacturing Example **5-1-1*** **APX2039**

4-((5-(2-aminopyridin-3-yl)isoxazol-3-yl)methyl)phenol (80mg, 0.30mmol) was dissolved in DMF (1 mL) and potassium 2-methylpropane-2-olate (1M in THF, 0.33 mL, 0.33 mmol) was added dropwise. The mixture was stirred for 5 min and a solution of 2,6-difluoropyridine (48.2 mg, 0.42 mmol) in DMF (0.5 mL) was added. The resulting mixture was stirred for 3min at 90°C and directly purified by flash chromatography to yield 3-(3-(4-((6-fluoropyridin-2-yl)oxy)benzyl)isoxazol-5-yl)pyridin-2-amine (65 mg, 0.18 mmol, 60%) as a white solid. MS: 363.3 $[M+H]^{+}$. ^{1}H NMR ($CDCl_3$) δ 8.13 (dd, J = 5.1, 1.7 Hz, 1H), 7.80 – 7.71 (m, 2H), 7.35 – 7.28 (m, 2H), 7.15 – 7.08 (m, 2H), 6.75 – 6.71 (m, 2H), 6.61 (dd, J = 7.9, 2.6 Hz, 1H), 6.30 (s, 1H), 5.55 (s, 2H), 4.07 (s, 2H), 2.32 (*b*s, 2H).

Fig S2. ^{1}H NMR spectrum of APX2039 in $CDCl_3$.

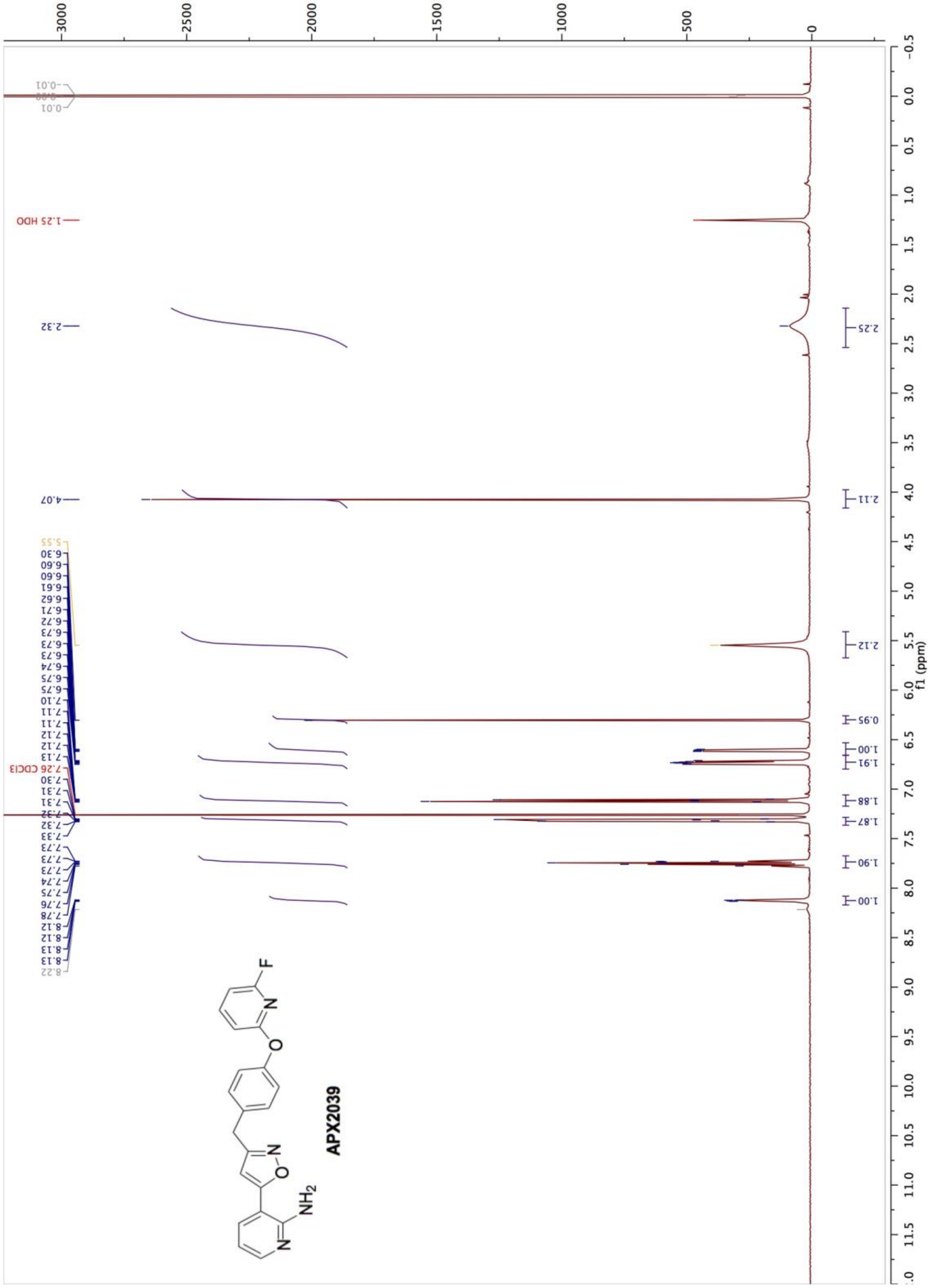

Fig. S3. Synthesis of APX2096 (2-amino-3-(3-(4-((6-fluoropyridin-2-yl)oxy)benzyl)isoxazol-5-yl)pyridin-1-ium-1-yl)methyl hydrogen phosphate (prodrug of APX2039)

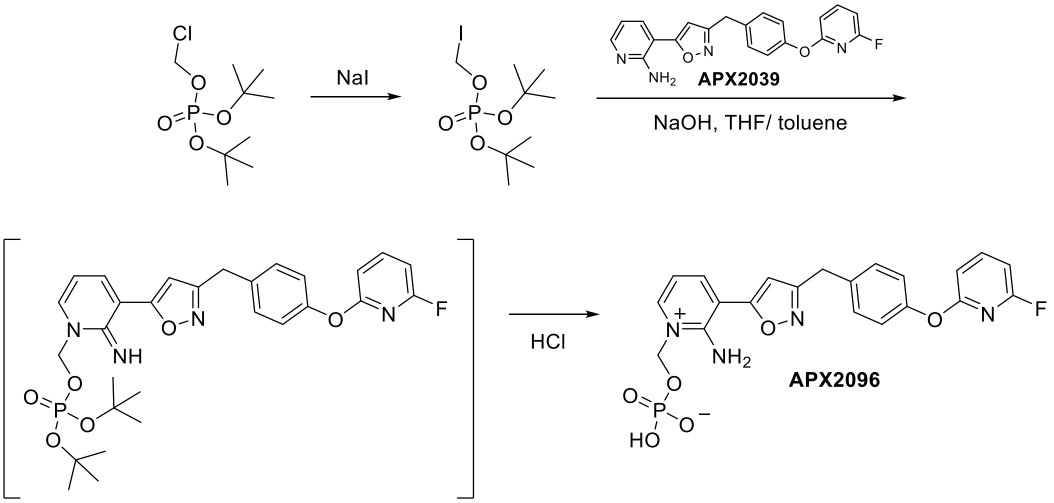

Sodium iodide (2.379g, 15.87mmol) and N-ethyl-N-isopropylpropan-2-amine (0.205 g, 1.587 mmol) were added to THF (7 mL). Di-tert-butyl (chloromethyl) phosphate (2.463 g, 9.52mmol) was added and the mixture was stirred at 45°C for 1.5 h. 3-(3-(4-((6-fluoropyridin-2-yl)oxy)benzyl)isoxazol-5-yl)pyridin-2-amine (2.3 g, 6.35 mmol) and toluene (7 mL) were added followed by the addition of sodium hydroxide (5N, 7 mL, 35 mmol). The mixture was stirred at 45°C for 30 min and then for 1h at 23°C. Another batch of freshly prepared iodomethyl phosphate (following the same procedure as described above, but using 1.2 g sodium iodide, 0.1 g N-ethyl-N-isopropylpropan-2-amine and 1.25 g di-tert-butyl (chloromethyl) phosphate in 4 mL of THF) was added and the mixture was stirred at 23°C for 12h. The organic layer was separated and the aqueous phase extracted three times with 5 mL of mixture of THF/toluene (1:1). The combined organic layers were cooled to 0°C and 5N HCl (7 mL) was added. The mixture was stirred at 23°C for 2 h. Water, ice and EtOAc were added and the pH of the aqueous phase was adjusted to 8-10.

The aqueous layer was extracted 3 times with EtOAc and then its pH was adjusted to about 7 to 7.5. The mixture became slightly cloudy during this process, but no significant precipitation occurred. The mixture was now filtered through a plug of reversed phase C18 silica gel (or alternatively a 0.45 micrometer PTFE filter). The pH of the clear filtrate was adjusted slowly to 4-5, while precipitation of the product occurred. The mixture was stirred for about 1.5 h and the product collected by filtration. The filter cake was thoroughly washed with water and 1x with a small amount of MeOH and finally dried under vacuum to give (2-amino-3-(3-(4-((6-fluoropyridin-2-yl)oxy)benzyl)isoxazol-5-yl)pyridin-1-ium-1-yl)methyl hydrogen phosphate as a beige powder (1.45 g, 3.07 mmol, 48% yield). MS: 473.4 $[M+H]^+$. ^{31}P NMR (DMSO-d_6/D_2O) δ 3.60 (s). ^1H NMR (DMSO-d_6/D_2O) δ 7.83 (q, *J* = 8.1 Hz, 1H), 7.73 (d, *J* = 7.0 Hz, 2H), 7.34 – 7.27 (m, 2H), 7.03 – 6.96 (m, 2H), 6.76 (s, 1H), 6.75 – 6.67 (m, 2H), 6.33 (t, *J* = 7.0 Hz, 1H), 5.39 (d, *J* = 7.4 Hz, 2H), 3.99 (s, 2H).

Fig. S4. ^{1}H NMR spectrum of APX2096 in DMSO-d_6/D_2O.

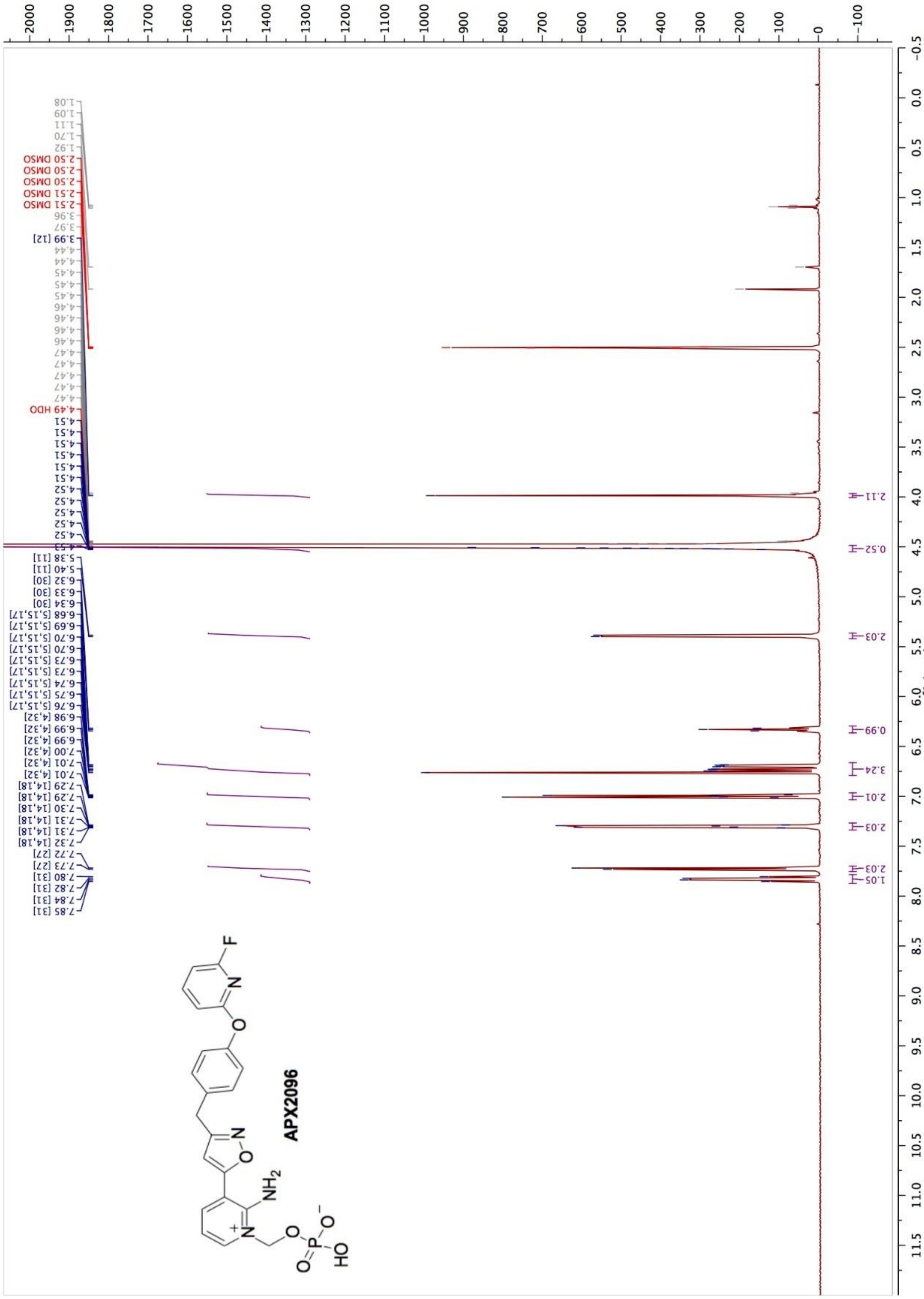

Fig. S5. Synthesis of APX2097 (2-amino-3-(3-(4-(benzyloxy)benzyl)isoxazol-5-yl)pyridin-1-ium-1-yl)methyl hydrogen phosphate (prodrug of APX2020) *(S1)

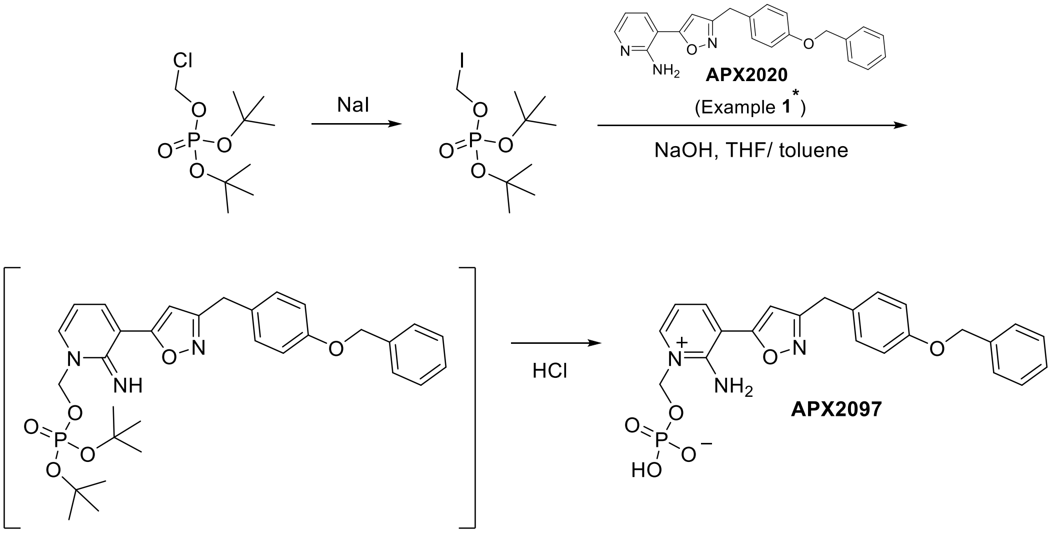

APX2097 was prepared according to the procedure for compound **APX2096** using Sodium iodide (944mg, 6.3mmol), N-ethyl-N-isopropylpropan-2-amine (81 mg, 0.63 mmol), di-tert-butyl (chloromethyl) phosphate (977 mg, 3.78 mmol), 3-(3-(4-(benzyloxy)benzyl)isoxazol-5-yl)pyridin-2-amine (900mg, 2.52mmol) and sodium hydroxide (5N, 2.77 mL, 13.85 mmol) to give (2-amino-3-(3-(4-(benzyloxy)benzyl)isoxazol-5-yl)pyridin-1-ium-1-yl)methyl hydrogen phosphate as a beige powder (390 mg, 0.83 mmol, 33% yield). MS: 468.4 $[M+H]^+$. ^{31}P NMR ($DMSO-d_6/D_2O$) δ 3.21 (s). ^1H NMR ($DMSO-d_6/D_2O$) δ 7.63 – 7.58 (m, 2H), 7.35 – 7.24 (m, 5H), 7.18 – 7.15 (m, 2H), 6.91 – 6.88 (m, 2H), 6.73 (s, 1H), 6.08 (t, J = 6.1 Hz, 1H), 5.28 (d, J = 5.3 Hz, 2H), 5.00 (s, 2H), 3.88 (s, 2H).

69 **Fig. S6. ^{1}H NMR spectrum of APX2097 in DMSO-d_6/D_2O.**

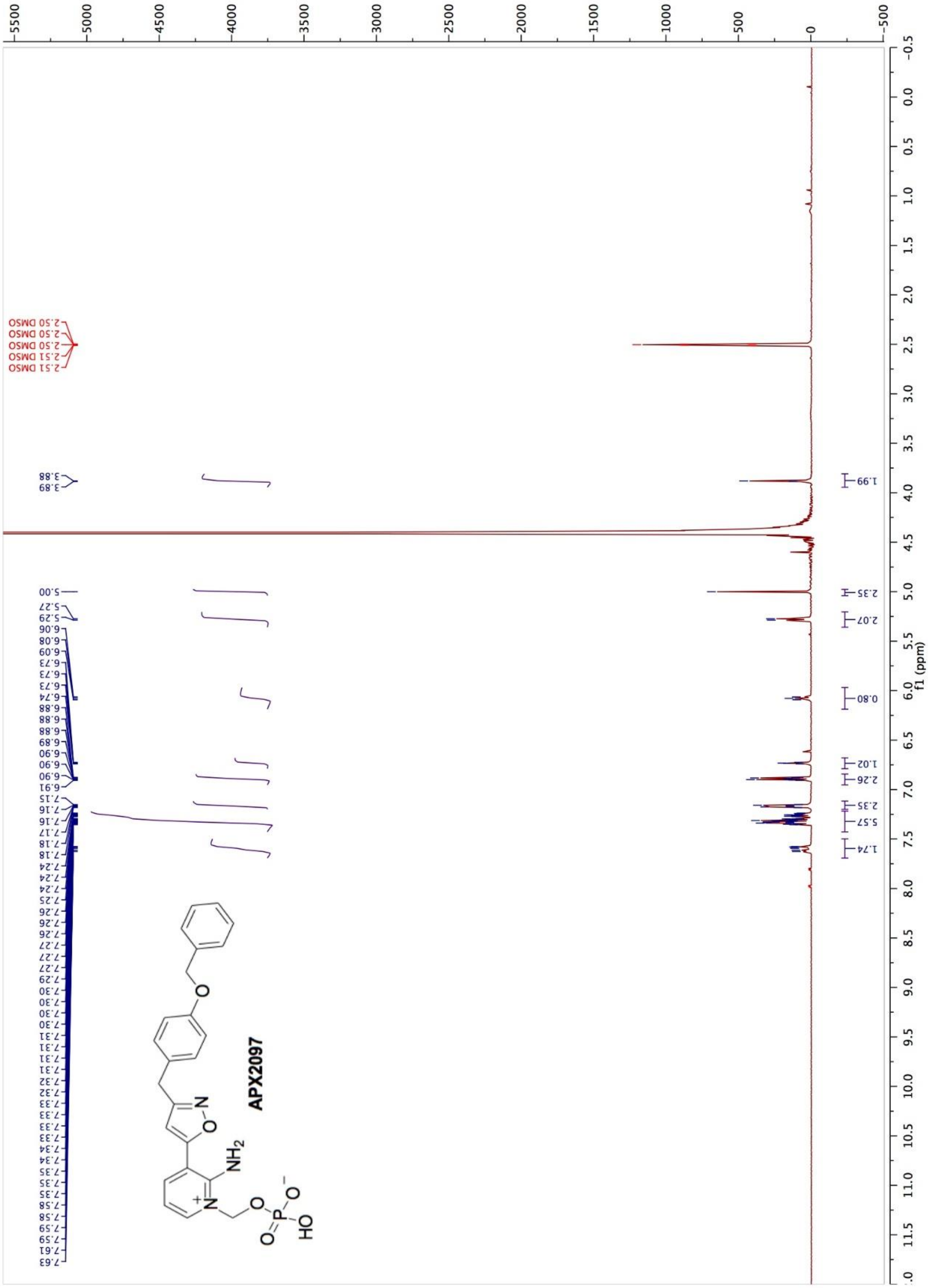

70

REFERENCES SUPPLEMENTARY MATERIAL

S1) Tanaka K, Inoue S, Murai N, Matsukura M, Nakamoto K, Shirotori S, Abe S. 2010. Heterocycles substituted pyridine derivatives and antifungal agent containing thereof. Patent 7,691,882 US, April 6, 2010.
